# Acceleration of epithelial cell syndecan-1 shedding by anthrax hemolytic virulence factors

**DOI:** 10.1186/1471-2180-6-8

**Published:** 2006-02-07

**Authors:** Taissia G Popova, Bryan Millis, Chris Bradburne, Svetlana Nazarenko, Charles Bailey, Vikas Chandhoke, Serguei G Popov

**Affiliations:** 1 National Center for Biodefense and Infectious Diseases, George Mason University, Manassas, VA 20110, USA

## Abstract

**Background:**

It has been recently reported that major pathogens *Staphylococcus aureus *and *Pseudomonas aeruginosa *accelerate a normal process of cell surface syndecan-1 (Synd1) ectodomain shedding as a mechanism of host damage due to the production of shedding-inducing virulence factors. We tested if acceleration of Synd1 shedding takes place *in vitro *upon treatment of epithelial cells with *B. anthracis *hemolysins, as well as *in vivo *during anthrax infection in mice.

**Results:**

The isolated anthrax hemolytic proteins AnlB (sphingomyelinase) and AnlO (cholesterol-binding pore-forming factor), as well as ClnA (*B. cereus *homolog of *B. anthracis *phosphatidyl choline-preferring phospholipase C) cause accelerated shedding of Synd1 and E-cadherin from epithelial cells and compromise epithelial barrier integrity within a few hours. In comparison with hemolysins in a similar range of concentrations, anthrax lethal toxin (LT) also accelerates shedding albeit at slower rate. Individual components of LT, lethal factor and protective antigen are inactive with regard to shedding. Inhibition experiments favor a hypothesis that activities of tested bacterial shedding inducers converge on the stimulation of cytoplasmic tyrosine kinases of the Syk family, ultimately leading to activation of cellular sheddase. Both LT and AnlO modulate ERK1/2 and p38 MAPK signaling pathways, while JNK pathway seems to be irrelevant to accelerated shedding. Accelerated shedding of Synd1 also takes place in DBA/2 mice challenged with *Bacillus anthracis *(Sterne) spores. Elevated levels of shed ectodomain are readily detectable in circulation after 24 h.

**Conclusion:**

The concerted acceleration of shedding by several virulence factors could represent a new pathogenic mechanism contributing to disruption of epithelial or endothelial integrity, hemorrhage, edema and abnormal cell signaling during anthrax infection.

## Background

It has been recently reported that major pathogens *Staphylococcus aureus *and *Pseudomonas aeruginosa *exploit acceleration of syndecan (Synd) ectodomain shedding as a mechanism of host damage and thus increase their virulence [1-3]. Approximately 1% of cell surface proteins are thought to be shed into the extracellular environment by host cells as a process of normal cell surface turnover [4], however during the infectious process shedding can reach pathological proportions. The diverse list of proteins, which are normally shed from the cell surface includes but is not limited to cytokines, growth factors, and cell adhesion molecules, such as tumor necrosis factor (TNFα), transforming growth factor α (TGFα), epidermal growth factors (EGFs), L-selectin, CD44, and Synds [[5] and citations within].

Synds are a group of four distinct proteoglycans (PGs), containing transmembrane core proteins modified with several heparan sulfate (HS) and chondroitin sulfate chains. Synd1 is the major HS PG of epithelial cells, which binds and regulates a wide variety of biological molecules through its HS chains [6]. Syndecans act as adhesion molecules, modulators of growth factor function, and co-receptors in processes as diverse as morphogenesis, tissue repair, host defense, tumor development, and energy metabolism. In mammary epithelial cells Synd1 co-localizes with actin filaments thus anchoring the cytoskeleton to extracellular matrix. Synd4 can be found in the focal adhesion junctions between cells [7]. Therefore, Synds are involved in modulation of cell spreading, adhesion, motility and maintenance of intercellular contacts. Ectodomain shedding rapidly changes the surface phenotype of affected cells and generates soluble, biologically active ectodomains that can function as paracrine or autocrine effectors. A growing body of evidence indicates that these molecular and cellular features enable ectodomain shedding to regulate many pathophysiological processes, such as microbial pathogenesis, inflammation, and tissue repair [1,8].

Shedding of syndecans can be abnormally increased in the case of the infectious process. The *P. aeruginosa *shedding enhancer was identified as LasA, a known metalloprotease virulence factor [1]. Studies *in vivo *indicate that *P. aeruginosa *activates Synd1 shedding to enhance its virulence in a murine model of lung infection [2]. Shedding enhancers of *S. aureus *are represented by pore-forming α-toxin and sphingomyelinase β-toxin [3]. During the infectious process, proteolytic removal of ectodomain in a soluble form by secreted microbial factors could enhance host colonization by altering the morphology and compromising the integrity of protective barriers formed by polarized epithelial cells of the skin, the surfaces of body cavities and internal organs, as well as endothelial cells lining blood vessel walls. The initial pathology can be further aggravated by exposing intercellular, basolateral, and subepithelial adhesive components to bacterial factors [9]. Structural damage to the host cell surface with resulting insult to protective barriers caused by ectodomain shedding along with pathological signaling can initiate a mechanism ultimately leading to the malfunction and failure of life-critical organs and systems.

Inhalation anthrax is a systemic disease characterized by severe damage to epithelia residing in major internal organs such as the liver, lung, intestines, spleen, and kidneys. Disruption of vasculature resulting in massive hemorrhages and pleural edema is a hallmark of systemic anthrax [10-12]. The *B. anthracis *genome contains genes for several proteolytic and hemolytic factors, which are structurally similar to the shedding inducers from *P. aeruginosa *and *S. aureus*, including among others the *S. aureus *α- and β-toxin homologues: anthralysin O (AnlO, pore-forming cholesterol-dependent hemolysin) and anthralysin B (AnlB, sphingomyelienase), respectively [13,14]. Another anthrax hemolytic factor of interest regarding its potential activity in ectodomain shedding is anthralysin A (AnlA, phosphatidyl choline-preferring phospholipase C, PC-PLC), which is 99% homologous to its *B. cereus *counterpart, cereolysin A (ClnA) [13]. Johansen *et al*. [15] reported that NIH 3T3 cells stably transfected with the gene encoding ClnA displayed a transformed phenotype. Exogenously applied ClnA decreased cell-cell contacts and increased cell migration [16]. Despite these observations the ectodomain shedding has never been studied with regard to infections caused by *B. anthracis *or *B. cereus*. Therefore, the main goal of this study was to test our hypothesis regarding the shedding activity of *B. anthracis *hemolytic proteins and to demonstrate that Synd ectodomain shedding takes place in response to anthrax infection.

In addition to the hemolysins our attention was attracted to the lethal toxin (LT), a major anthrax virulence factor [17]. The mediator(s) of its toxicity remains unknown. It has been shown that LT abrogates intracellular signaling through proteolytic cleavage of mitogen-activated protein kinase kinases (MAPKK) [18]. Administration of lethal doses of a purified LT to mice and rats causes pleural edema [19] indicating that LT compromises the integrity of intercellular contacts maintaining the fluid homeostasis in the lung. It has been previously suggested that LT is capable of increasing vascular permeability [20], and recent experiments with endothelial cells in culture are consistent with this conclusion [21]. Therefore, we wanted to test whether LT can function as a shedding inducer, and whether the inhibition of MAPKKs by LT modulates cell signaling relevant to shedding.

In the current study, we report characterization of the isolated anthrax hemolytic proteins AnlO, AnlB, as well as the AnlA homolog ClnA from *B. cereus*, regarding their Synd shedding and barrier permeability-enhancing activities. We found that LT also stimulates Synd1 shedding (although to a less extent compared to hemolysins) by the mechanism which involves the MAPKK signaling pathways. Overall, our observations identify a novel strategy of anthrax virulence factors, which among their diverse biochemical activities stimulate the host cell stress responses ultimately leading to activation of the ectodomain shedding. In connection with these observations we also demonstrate that Synd1 shedding occurs during the infectious process in the *B. anthracis *(Sterne) spore-challenged mice. The elevated levels of shed Synd1 are readily detectable in the circulation just 24 h post challenge. This new feature of the anthrax infectious process could be of high pathogenic significance. We suggest that a cumulative effect of several virulence factors on ectodomain shedding could compromise the integrity of host protective barriers, cause malfunction of major organs, and ultimately contribute to the pathological systemic response typical in anthrax patients.

## Results

### Hemolytic factors and LT increase ectodomain shedding from normal murine mammary gland (NMuMG) epithelial cells

**Figure 1:**
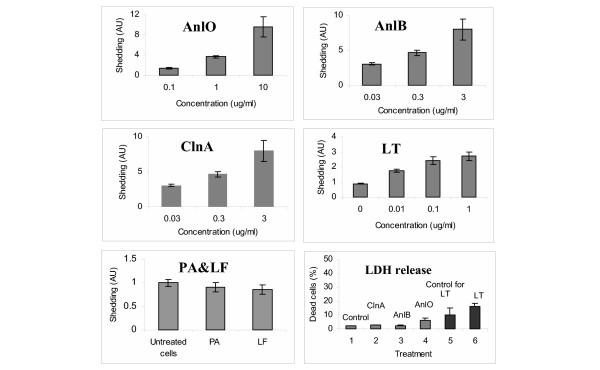
Synd1 shedding from NMuMG cells after treatment with *B. anthracis *proteins in the presence of 1% FCS for 4 h (24 h in the case of LT, PA and LF). The lower right panel shows cell death measured after 4 h as LDH release for untreated cells (1), 1 μg/ml ClnA (2), 1 μg/ml AnlB (3), 0.1 μg/ml AnlO (4), untreated cells after 24 h (5), 1 μg/ml LT after 24 h (6). Error bars represent 95% confidence intervals (n = 6).

**Figure 2:**
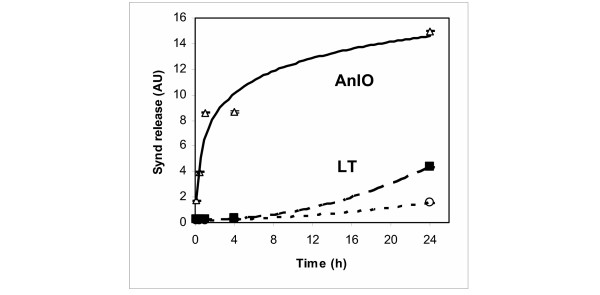
Time course of Synd1 release from NMuMG cells transferred into 1% FCS media and challenged with 1 μg/ml of either AnlO (triangles), or LT (squares). Control cells were left untreated (circles). Each measurement corresponds to average of three culture wells. Three independent experiments have been performed with similar results.

Shedding activity of isolated recombinant proteins identified by us as candidate shedding inducers was tested using NMuMG epithelial cells. This murine cell type represents a commonly used model in similar studies [3]. Synd1 is abundant on the epithelial cells and can be assayed using antibodies specific to the corresponding core proteins or the HS chains. Using immunodetection with antibodies specific for Synd1 core protein we tested NMuMG cell culture supernatants for the presence of soluble Synd1 in the assay conditions when highly charged HS chains are selectively retained by the blotting membrane. We found that all tested hemolytic proteins, namely AnlO, AnlB and ClnA, as well as LT cause a concentration-dependent acceleration of Synd1 release into culture supernatants albeit at significantly different amounts (Fig. 1). The time course of Synd1 release in the case of AnlO shows a fast increase in the amount of shed Synd which continues for several hours (Fig. 2). The LT causes increase in Synd1 shedding, although the effect of LT is of lower intensity and develops slower compared to AnlO. The individual components of LT, the protective antigen (PA) and the lethal factor (LF) do not induce shedding (Fig. 1). This suggests that neither the extracellular enzymatic activity of LF nor the sole pore-forming capacity of PA is directly responsible for shedding.

Analysis of cell viability in the shedding experiments reveals a marginal to small degree of the lactate dehydrogenase (LDH) release from treated cells compared to untreated ones. There is no obvious correlation of cell death with the amount of shed Synd1, which allows us to conclude that the processes of shedding and cell death are not directly related to each other but rather take place concomitantly, depending on the nature of the pathogenic factor and other treatment conditions. For example, treatments with either ClnA or AnlB presented in Fig. 1 are not cytolytic, while the amounts of shed Synd1 in both cases increase 8-fold. AnlO increases cell death 3-fold (from 2% to 6%), while there is an 11-fold increase in Synd1 shedding. In the case of LT, incubation for 24 h leads to a 1.6-fold increase in cell death (from 10% to 16%), while the amount of shed Synd1 increases almost 3-fold. The above conclusion agrees with direct microscopic observation of treated monolayers displaying live cells with partially or completely shed Synd1 detected by fluorescently-labeled anti-Synd1 antibodies (see below). We did not explore this topic further, however available data show that the E-cadherin shedding preceded apoptosis in enterocytes [22], while in endothelial cells LT compromised the barrier integrity (presumably through ectodomain shedding) independently of apoptosis or necrosis [21]. Stimulation with the phorbol ester, PMA, a known inducer of shedding [5], prolonged viability of epithelial cells [23] and predisposed monocytes to apoptotic death caused by LT [24].

**Figure 3:**
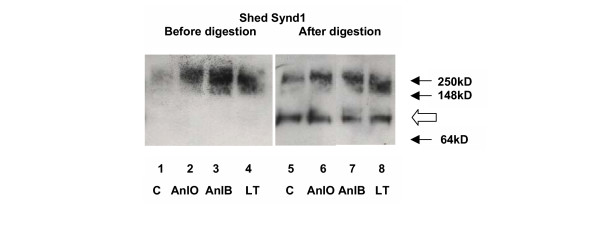
Western blot of Synd1 shed from NMuMG cells by *B. anthracis *proteins in presence of 1% FCS for 24 h. Synd1 from culture supernatants was partially purified using DEAE ion exchange chromatography and analyzed before (lanes 1 to 4) and after (lanes 5 to 8) partial digestion for 5 h with both heparanase II (10 mU/ml) and chondroitin sulfate ABC lyase (20 mU/ml) to remove glycosaminoglycan chains. For digestion the amount of total protein in each reaction was adjusted approximately equal. Gel lanes correspond to untreated cells (1, 5) and cells challenged with: 0.1 μg/ml of AnlO for 4 h (2, 6); 1 μg/ml of AnlB for 4 h (3, 7); 1 μg/ml of LT for 24 h (4, 8). Synd1 core protein appears as a band of approximately 80 kD (open arrow).

In order to confirm the biochemical identity of shed Synd1, the following experiment has been carried out. Western blot of NMuMG cell supernatants after treatment with either AnlO, AnlB or LT using anti-mouse Synd1 antibody demonstrates a high molecular mass smear band that can be attributed to the presence of heterogeneous heparan sulfate glucosaminoglycan chains in shed Synd1 (Fig. 3). Indeed, digestion of the supernatants with heparanase II and chondrotin sulfate ABC lyase leads to the appearance of a single band corresponding in gel mobility to the core Synd1 protein, which typically migrates in a gel as an approximately 80 KD band [3].

### Immunostaining of the NMuMG monolayers with fluorescently-labeled antibodies against E-cadherin and Synd1

**Figure 4:**
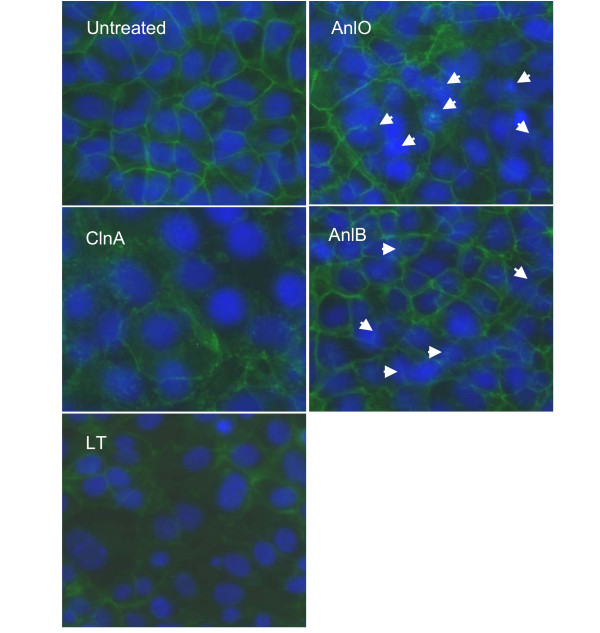
Immunohistochemical detection of E-cadherin in monolayers of NMuMG cells after treatment with LT and hemolytic proteins (1 μg/ml each for 16 h). Nuclei are stained blue with DAPI, and E-cadherin is stained green with FITC-conjugated anti-E-cadherin antibody. In the case of AnlO and AnlB, arrows indicate some of the most damaged areas. 40× magnification.

**Figure 5:**
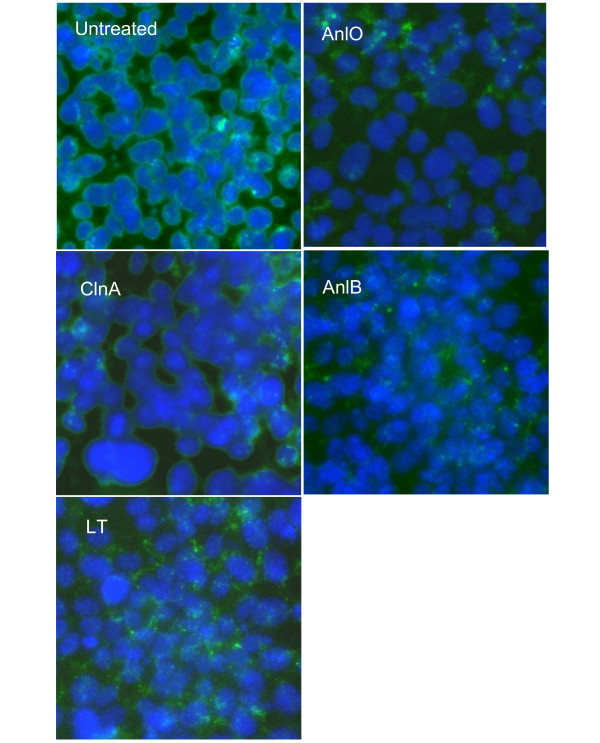
Immunohistochemical detection of Synd1 in monolayers of NMuMG cells after treatment with LT and hemolytic proteins (1 μg/ml each for 16 h). Nuclei are stained blue with DAPI, and Synd1 is stained green with FITC-conjugated anti-Synd1 antibody. 40× magnification.

We examined the NMuMG cells grown on glass slides using immuno-fluorescence microscopy. After challenge with proteins causing Synd1 shedding the cells were fluorescently stained using E-cadherin- and Synd1-specific FITC-conjugated monoclonal antibodies. E-cadherin is a major transmembrane component of the apical junctional complex. In a simple polarized epithelium the junctional complex consists of tight junctions and underlying adherens junctions playing a key role in the formation and maintenance of epithelial barriers [25]. We therefore wanted to see if the process of Synd ectodomain shedding observed in our experiments is accompanied by the loss of intercellular contacts reflected in the dissociation of the junctional complex. Our analyses revealed obvious cytopathogenic changes in treated NMuMG cells. In all cases, a network of E-cadherin visible in untreated confluent cells becomes either disorganized, damaged or disappears from intercellular contacts upon treatment (Fig. 4), similar to what has been reported for VE-cadherin in endothelial cells treated with LT [21]. Partially confluent NMuMG cells demonstrate intensive Synd1 staining along the perimeter of cells, which partially or completely disappears from cell surfaces after incubation with the shedding-inducing proteins, while remnants of the ectodomain remain visible in the intercellular space (Fig. 5). Notably, the treated cells retain a high intensity of DAPI blue fluorescence typical for undamaged nuclei, indicating that the loss of E-cadherin and Synd1 takes place from viable cells.

### Inhibition of Synd1 release

The ectodomain shedding of cell surface molecules is typically mediated by host metalloproteinase sheddases [5,26]. Both constitutive and accelerated shedding are inhibited by a variety of substances active in a number of receptor- and stress-activated signaling pathways, which involve protein tyrosine kinases (PTKs), protein kinase C (PKC), and mitogen-activated protein kinases (MAPKs) [3,5,26]. The activity of LT in macrophages and epithelial cells has been previously reported to involve down regulation of MAPK kinase cascades [18,23].

The results of inhibition experiments are presented in the Table. It shows that piceatannol, a specific inhibitor of the Syk family of PTKs [27] is active in both spontaneous and induced Synd1 shedding for all tested proteins. In the case of AnlO and LT at low concentration of 0.5 μM the inhibitor shows some stimulatory effect on both constitutive and induced shedding, but it strongly inhibits Synd1 release in concentrations typical for its activity range of 5 to 50 μM [28]. The effect of piceatannol suggests that all four factors stimulate signaling pathways, which most probably involve cytoplasmic Syk, however piceatannol has also been reported to inhibit other tyrosine kinases in a similar concentration range. In agreement with the above suggestion the inhibitor of Src PTK family PP2 is completely inactive (data not shown). A general PTK inhibitor tyrphostin A25 (at 0.5 to 5 μM) shows only a weak activity. The phosphatidylinositol-3-kinase inhibitor LY294002 is inactive with all tested proteins, and we conclude that the cell survival pathway mediated by this kinase is irrelevant to shedding.

Suramin is a multi-potent therapeutic [29], which among other activities displays an antitumoral effect by blocking the growth factors binding to several receptors, including the ones for epidermal growth factor (EGF), platelet derived growth factor (PGDF), insulin growth factor II, and transforming growth factor-β (TGF-β). These growth factors bind to heparan sulfate-containing proteoglycans, which can be shed in various pathophysiological processes, such as wound repair, and microbial infections [26,30]. Most importantly, suramin modulates activity of protein tyrosine phosphatases (PTPs) involved in cell adhesion, integrin signaling and cell cycle progression [31,32]. Among these, PTP1B and Cdc25A are inhibited by suramin in the low μM range. The drug activates PTPα and PTP LAR at higher concentrations. Because of low bioavailability, the suramin concentration above 50 μM has to be used for the activation effect [33]. The Table shows that similar to piceatannol, suramin stimulates shedding at 20 μM. At higher concentration, suramin effectively inhibits Synd1 shedding in NMuMG cells induced by LT and AnlO. This effect is consistent with the inhibition-activation pattern of suramin activity toward PTPs, but the multi-potency of suramin excludes its clear interpretation without additional studies. Shedding activities of lipases ClnA and AnlB are insensitive to suramin at all concentrations tested.

In order to understand which signaling pathways among p38, ERK and JNK are involved in LT-mediated acceleration of Synd shedding, we tested SB202190, an inhibitor of p38; PD98059, an inhibitor of MEK1/2 (ERK pathway); and the JNK inhibitor II. While both AnlO and LT induce Synd1 shedding, LT on itself is a known inhibitor of MAPK signaling. In contrast, AnlO was reported to stimulate p38 in macrophages [34]. We found that the p38 inhibitor in the range of 1 to 10 μM decreased Synd1 shedding in NMuMG cells induced by either AnlO or LT to the level of spontaneous shedding observed in cultures without treatment, but it is inactive with ClnA and AnlB. The inhibitor of ERK pathways PD 98059 (at 5 to 50 μM) behaves similar to SB202190 with AnlO, but it is less effective in the LT-induced shedding. The only statistically reliable effect of the JNK inhibitor is the slight increase in spontaneous shedding from untreated cells. None of the tested inhibitors is toxic to cells in the conditions of the inhibitor experiments (viability of inhibitor-treated control cells remains at the level above 90%, data not shown) indicating that activity of tested inhibitors is not dependent on their cytotoxic effect.

Collectively, the inhibition experiments demonstrate that *B. anthracis *pathogenic factors induce Synd1 shedding through different signaling pathways, which seem to converge on activation of cytoplasmic PTKs. Among tested proteins, one can preliminarily identify two groups of shedding inducers. The first one includes LT and pore-forming AnlO influencing MAPK pathways, which are commonly activated in response to receptor stimulation and stress, while the other consists of membranolytic lipases ClnA and AnlB.

### ERK1/2 and p38 phosphorylation patterns in NMuMGs treated with AnlO or LT

**Figure 6:**
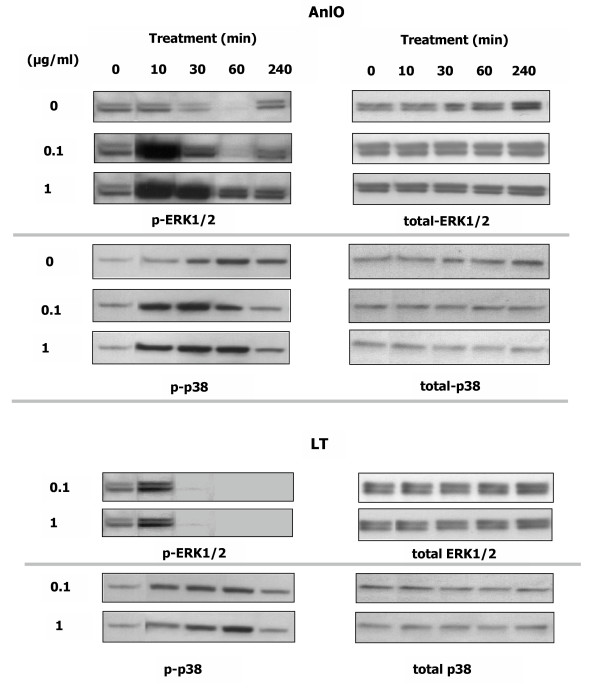
AnlO and LT cause transient phosphorylation of ERK1/2 and p38 in NMuMG epithelial cells. Cells were transferred into growth media with 1% FCS, treated with indicated concentrations of either AnlO or LT for different periods of time and lysed. Western blots were probed with phosphorylation-specific antibodies against ERK1/2 and p38. Results from control untreated cells are shown in the AnlO panel.

Since both PD98059 and SB202190 influence the AnlO- and LT-induced Synd1 shedding, ERK1/2 and p38 phosphorylation patterns were studied in more detail. Fig. 6 shows that, compared with untreated cells, within several minutes the AnlO causes a strong transient ERK1/2 activation lasting for more than 4 h in the presence of 1 μg/ml AnlO. A lower concentration of AnlO causes a shorter period of activation followed by a slight increase in signaling after 4 h. The latter is present in both control and treated cells and probably reflects a distress caused by incubation in low FCS media. The p38 phosphorylation reaches maximum intensity after the ERK1/2. In the same conditions, LT also causes transient activation signals but its effect on the amounts of activated ERK1/2 and p38 is different from that of AnlO: the up-regulation of ERK1/2 phosphorylation is detectable at the 10 min time point and then completely disappears within 30 min. While the transient peak of p38 activation is detectable at 0.1 μg/ml of LT, its level remains lower compared to AnlO. As a control, LT (1 μg/ml) boiled for 15 min is inactive in MAPK activation (not shown). It seems that the enzymatic cleavage of MAPKKs by LT is an important factor reducing the intensity of signaling.

### Anthrax LT and hemolytic factors compromise epithelial barrier permeability

**Figure 7:**
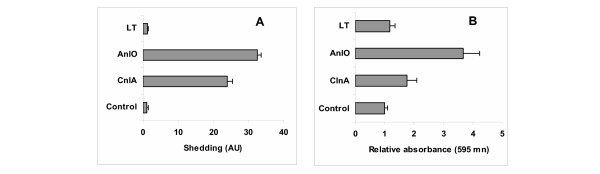
Shedding of Synd1 into culture supernatants (A) and changes in barrier permeability (B) for HSAECs treated with the hemolytic proteins and LT. The cells initially confluent on a 12 mm insert (Costar, 3.0 μm pores) were treated with 1 μg/ml of each protein for 4 h in 1% FCS media. After treatment the change in barrier permeability was tested as increase in the absorbance of Blue Dextran 2000 added for 2 h to the culture media in the top chamber and detected in the lower chamber, in comparison with untreated cells. Error bars represent 95% confidence intervals; 3 culture plate wells were used for each measurement.

After our findings demonstrated the fact of accelerated Synd shedding, it was important to test if the latter is accompanied by changes in barrier permeability. We used a primary culture of human small airway epithelial cells (HSAECs) grown on collagen-coated membranes with the pores permeable to Dextran Blue 2000, which was used as an indicator of barrier integrity [35]. The membranes separated the lower and the upper chambers of the culture wells, thus mimicking the barrier represented in the lower airways of the lung. The cells were challenged by adding AnlO, ClnA or LT in the upper chambers for 4 h. After treatment, Blue Dextran 2000 was added to the upper chambers for 2 h. The changes in barrier permeability were evaluated by measuring the optical absorbance of Blue Dextran 2000 in the lower chambers, in comparison with untreated cells. Fig. 7 shows that the AnlO and ClnA cause intensive shedding of Synd1. In these experimental conditions the LT challenge does not induce shedding and therefore serves as a negative control. The effect of LT becomes detectable only after the 24 h (data not shown). In general, the HSAECs display the pattern of sensitivity to AnlO, ClnA and LT similar to that of the NMuMG cells. As expected, the treatment with AnlO and ClnA causes a strong increase in barrier permeability (Fig. 7B) correlating with shedding. This suggests a possible causal relationship between the two processes, which needs to be elaborated in further studies.

### Anthrax infection in mice is accompanied by acceleration of Synd1 shedding

**Figure 8:**
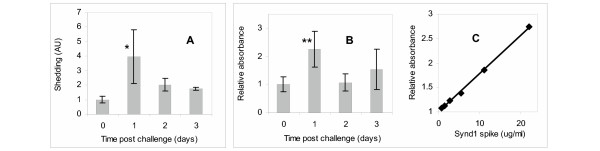
Synd1 content in sera of mice challenged with 30 LD_50 _of Sterne strain spores intraperitoneally. The serum samples were either dot-blotted in duplicates onto membrane (A) or were used to cover wells of the ELISA plate (B) for immunodetection of Synd1 with antibody 281-2. Graphs represent average fold increases over control. In (A), two mice were used at the day of challenge, three mice at each day 1 and 2, and two mice at day 3. In (B), seven mice were used at each of the days 0, 1 and 2, and three mice at day 3. A calibration concentration-response curve obtained by triplicate measurements of control serum spiked with recombinant Synd1 is shown in (C). The bars indicate 95% confidence intervals. *P < 0.05, **P < 0.01.

The experiments with recombinant proteins described above suggested that Synd shedding could also take place during anthrax systemic infectious process. To confirm this hypothesis the DBA/2 mice were challenged with *B. anthracis *spores of the toxigenic Sterne strain intraperitoneally as described before [36]. Blood samples were drawn, and serum was tested similar to culture supernatants of protein-treated cells using immunoblot with the antibody against Synd1 core protein. The assay shows a several-fold increase in the amount of shed Synd1 on the next day post challenge (Fig. 8A). ELISA protocol with the same antibody demonstrates similar results, when diluted serum samples are used to coat the assay plate wells (Fig. 8B). A high level of circulating ectodomain is sustained until two days post-infection. A calibration curve obtained with a control serum spiked with different concentrations of recombinant Synd1 allows estimate that average blood Synd1 concentration at day 1 post challenge is increased by 18 μg/ml.

In the conditions of the experiment, about 50% of animals die at day 3, and all animals die by day 6 post challenge. The onset of death on day 3 is accompanied by a decrease in the amount of released Synd1, which could be explained by a number of mechanisms, such as degradation of syndecan core protein resulting in the loss of its immunoreactivity with antibodies and a reduced retention of the protein on the surface of the assay membrane or plate. In any case, the abnormal release of Synd1 into circulation of infected mice directly indicates that the pathological acceleration of Synd1 shedding takes place *in vivo *at systemic level and is accompanied by the processes of its biochemical turnover.

## Discussion

Acceleration of ectodomain shedding represents a part of an adaptive response of the host cells to different stress factors and injury such as G protein-coupled receptor agonists, growth factors, cytokines, osmotic stress, wounding and phorbol ester activation [37-39]. However the functional significance of the ectodomain shedding in microbial pathology is uncertain: it could either promote pathogenesis, cellular defenses or both. Microbial membrane-damaging factors and other toxins can disturb cell homeostasis and serve as strong inducers of stress proceeding through activation of signaling pathways ultimately resulting in cytoskeletal rearrangements and increase in barrier permeability [40]. Although the cytoskeletal rearrangements and Synd1 ectodomain shedding are closely interconnected [6,41], a direct link between stress response, Synd1 ectodomain shedding and barrier dysfunction has never been demonstrated for bacterial toxins.

Initial evidence that *B. anthracis *toxins can disrupt host epithelial and endothelial barriers is available from early anthrax publications. For example, Smith *et al*. [42] using LT produced *in vivo *identified vascular damage and renal failure as a consequence of its activity, while Smith and Stoner [20] demonstrated that LT induced an increase in vascular permeability. These observations agree with the fact that the most damaged organs in the infectious process are the ones with high epithelial and endothelial cell content such as spleen, lungs, liver, renal system, vasculature of blood and lymphatic vessels. A recent report by Warfel *et al*. [21] confirmed that LT can increase the endothelial barrier dysfunction independent of necrosis or apoptosis. The LT preparations used in early studies were crude, so we took into account a possibility that pathogenic factors other than LT could have played role in the observed effects. A spectrum of these factors includes (but may not be limited to) cytolytic lipases and pore-forming toxins [13,43], and proteases of different specificity [14,36].

Our experiments demonstrate that bacterial secreted factors, such as pore-forming toxin AnlO, and cytolytic lipases ClnA and AnlB accelerate the normal process of host cell Synd1 and E-cadherin ectodomain shedding, which is as a proteolytic mechanism of releasing them in a soluble form [4]. The effects of AnlO and AnlB are similar to what has been previously reported for their biochemical analogs, staphylococcal α- and β-toxins, respectively, in a similar concentration range [3]. We further show that the process of Synd1 shedding is accompanied by loss of epithelial barrier integrity of HSAECs in culture. The abnormal level of shed Synd1 in the blood of spore-challenged mice suggests that anthrax secreted factors could compromise epithelial barrier integrity at the early stages of the disease.

The full spectrum of proteins shed in anthrax requires further studies. Our preliminary data (not shown) indicate that different combinations of shedding factors could produce synergictic effects. Biologically relevant concentrations of anthrax shedding inducers in tissues, organs and body fluids are unknown, and therefore the assessment of each protein's contribution to ectodomain shedding *in vivo *is currently impossible, but the capacity of *B. anthracis *to produce hemolytic proteins, in addition to LT, has been demonstrated in both aerobic and anaerobic culture conditions [13,43,44]. The antibodies against these proteins are also detectable in serum of mice challenged with *B. anthracis *(Sterne) spores (data not shown). In our experimental conditions the release of Synd1 and E-cadherin takes place within several hours, and high levels of shed Synds are detectable after 24 hours post infection with spores (we have not tested earlier time points). This observation opens a possibility of using shed ectodomain release into circulation for early detection of the anthrax infectious process. Maximal Synd release coincides in time with the appearance of bacteria in the spleens of challenged animals tested in our previous experiments [45]. Bacteria became detectable in the spleens at approximately 16 h post infection, reached maximum numbers at about 24 h and then declined before death.

Normally, ES is mediated by metalloproteinases, which are collectively called sheddases or secretases. Our data agree with the host sheddase modulation mechanism demonstrated by others [3,5,38], because metalloproteinase inhibitors such as galardin and phosphoramidon reduce shedding induced by AnlO (data not shown), which has no enzymatic activity on its own. Other anthrax proteins, ClnA and AnlB are not proteases, and therefore cannot shed Synd1 by direct proteolysis on the cell surface. LT is a metalloprotease but induction of Synd1 shedding requires LT delivery into the host cell, in agreement with the extracellular cleavage of the Synd1 core protein by cellular sheddase. The fact that proteins of an absolutely different nature, such as proteases and lipases of distinct enzymatic specificities along with pore-forming toxins possessing no catalytic activity, display similar effects with regard to Synd1 shedding indicates activation of a common intracellular mechanism by diverse extracellular signals. Indeed, the activity of piceatannol against shedding by all tested inducers suggests that cytoplasmic Syk PTK serves as the common point of convergence. This mechanism however retains a certain level of specificity judging by the fact that neither PA nor LF induces shedding.

The MAPK-mediated pathways have been previously implicated in receptor-induced ectodomain shedding [5]. It has also been reported that AnlO stimulated the p38 signaling in macrophages [34]. Our data show that the inhibitors of ERK1/2 (PD98059) and p38 (SB202190) decrease the AnlO- and LT-induced shedding. This effect agrees with the mechanism recently discovered for the hydrogen peroxide-stimulated cytoskeletal reorganization in endothelial cells [46]. It has been shown that both of the above inhibitors attenuated MAPK-mediated activation of the small heat shock protein Hsp27 downstream from ERK1/2 and p38. This protein is responsible for the actin stress fiber polymerization, which accompanies Synd ectodomain shedding [41,47,48]. We however cannot conclude which of the MAPK pathways plays a predominant role in shedding.

Currently available data suggest that the p38 pathway defends against bacterial pore-forming toxins *in vivo *and *in vitro *[49], therefore the p38-mediated shedding could represent a protective cell response to stress (unless it reaches a pathological proportion). In agreement with this, Kevil *et al*. [50] reported that p38 inhibitor attenuated the oxidant stress fiber formation and prevented generation of gaps between endothelial cells. Warfel *et al*. [21] found that p38 inhibitor increased the endothelial barrier resistance, although concluded that this effect was somewhat paradoxical given the ability of LT, as known inhibitor of MAPK signaling, to decrease barrier resistance. We suggest that LT plays a dual role upon interaction with the host cells by inducing both the cellular stress and the inhibition of MAPK activation. The intricate combination of both processes results in the reduction of the transient stress signal, which nevertheless remains sufficient for the induction of shedding but reduces the potency of LT as shedding inducer. In support of this, our data show that LT induces small but detectable activation of p38 even at high LT concentration. In contrast to LT the pretreatment of cells with the effective p38 inhibitor is expected to completely block the stress signal and the consequent shedding. Transient p38 activation has never been reported in connection with LT activity although LT-induced ERK1/2 activation has recently been described as toxin-induced LT resistance in macrophages [51] in a lower concentration range compared to our experiments.

## Conclusion

Our findings provide additional insights into the studies of *B. anthracis *virulence factors, such as LT and hemolytic proteins, at cellular and organism levels, where shedding has to be taken into account as a new anthrax pathogenic mechanism. It could rapidly change cell surface properties and increase barrier permeability by the concerted effect of several pathogenic factors contributing to dissemination of infection, hemorrhages and edema. Shedding generates biologically active ectodomains that can function as paracrine or autocrine effectors [8]. The shed soluble proteoglycans are highly hydrated and this effect is expected to exacerbate edema by causing influx of water into the intercellular space. It has already been shown that shed Synd1 is toxic to mice [52], and that inoculation of Synd1 ectodomain restored sensitivity of Synd^-/- ^mice to *P. aeruginosa *[2]. These findings suggest high pathological significance of Synd shedding in the anthrax infectious process, which needs to be further addressed in animal experiments.

## Methods

### Inhibitors, reagents and isolated proteins

NMuMG epithelial cells (CRL-1636) were from ATCC (Manassas, VA), human lung epithelial cells (HSAEC) were from Cambrex, Inc. (Walkersville, MD). DMEM media was from ATCC (Manassas, VA), other cell culture reagents were from Cellgro (Herndon, VA). Galardin, tyrphostin A25, piceatannol, suramin, SB202190, PD98059, JNK inhibitor II and PP2 were from Calbiochem (Darmstadt, Germany). Anti-human Synd1 antibody clone Mi15 and rat anti-mouse Synd1 (clone 281-2) were from BD Biosciences (San Diego, CA). Antibody against total and double phosphorylated p38 (Thr180/Tyr182), ERK (Thr202/Tyr204) and JNK (Thr183/Tyr185) were from Cell Signaling Technology (Beverly, MA). Hemolytic *B. anthracis *proteins were expressed in *E. coli*, purified and characterized as described before [13]. They are at least 95% homogeneous based on the results of SDS-PAGE analyses. The phospatidyl choline-preferring phospholipase C from *B. cereus *(cereolysin A) was purchased from Sigma (MA) and was used without further purification. Recombinant protective antigen (PA) and lethal factor (LF) were purchased from List Biological Laboratories (Campbell, CA). The endotoxin content of all proteins was determined by Quantitative Chromatogenic LAL kit (Cambrex, MD). Recombinant murine Synd1 expressed in *E. coli *as a His6-tagged protein (>95% purity) was a gift from Prof. Myung-Chul Chung (George Mason University, VA). The protein concentration was determined using Bradford assay with BSA as a standard.

### Activation of shedding in cultured cells

Human Small Airway Epithelial Cells, or HSAECs (Cambrex, Inc., Walkersville, MD), were grown in DMEM/F12 complete medium with 10% fetal calf serum (FCS, Gibco). Before challenge the FCS content was reduced to 1%. NMuMG cells were grown up in Dulbecco's modified Eagle's medium with 4.5 g/l glucose, 10 μg/ml insulin, and 10% FCS. HSAECs were grown up in Ham's F12 media supplemented with non-essential aminoacids, pyruvate, β-mercaptoethanol and 10% FCS. Cells were seeded in 96-well plates, cultured to 1 day post confluence, then stimulated with indicated proteins using serum-free media from Cellgro (Herndon, VA) supplemented with 1% FCS. LT was used as a mixture of equal amounts of PA and LF at total concentration of 1.0 μg/ml, 0.1 μg/ml, and 0.01 μg/ml. After stimulation, the plates were spun down at 1,100 × g for 10 min and supernatant was frozen at -20°C for further analyses. In control experiments, a known inducer of Synd1 shedding, PMA (4α-phorbol-12,13 didecanoate) in concentration 10 μM in 1% FCS media induced 4-fold increase in shed Synd-1 from NMuMG cells after 24 h incubation. In the same conditions, endotoxin in concentration 10 ng/ml induced neither significant shedding nor activation of p38 and ERK1/2. Pre-treatment of AlnO (1 μg/ml) with polymyxin (50 μg/ml) had no effect on the amount of shed Synd1 and the p38 or ERK1/2 phosphorylation. Endotoxin contamination from added proteins in shedding experiments did not exceed 5 ng per ml of culture medium.

### Dot Blot Assays

Supernatant from treated cells (100 μl) was added to 1 ml of acidification buffer (150 mM NaCl, 50 mM NaOAc, 0.1% Tween-20, pH 4.5). Immobilon NY+ membrane (9 × 12 cm, Millipore) was prepared by first soaking it in acidification buffer. *Bio-Dot *microfiltration apparatus (Bio-Rad, CA) was used for all syndecan dot blots. The sample wells were re-saturated with 100 μl of acidification to prevent drying of the membrane. 400 μl of sample solution (in acidification buffer) was used per well on the apparatus. First the sample was kept in the wells on top of the membrane for 3 min and then it was drawn through the filter/membrane for another 3 min. The wells were rinsed twice with 400 μl of acidification buffer. The membrane was taken out, rinsed twice for 5 min in acidification buffer and blocked with 3% powdered milk in buffer #2 (150 mM NaCl, 10 mM Tris-HCl, pH 7.4) for 1 h. The membrane was then incubated with rat anti-mouse Synd1 antibody at a dilution of 1:1000 in 3% milk in buffer #3 (150 mM NaCl, 10 mM Tris-HCl, 0.3% Tween-20, pH 7.4) for 2 h on a platform shaker at room temperature. For the cells of human origin the antibody against human Synd1 has been used in a dilution 1:2000. The membrane was then washed with buffer #3 three times for 5 min and incubated with goat anti-rat HRP-conjugated secondary antibody at a dilution of 1:7500 for murine Synd1 blot, or with goat anti-mouse HRP-conjugated polyclonal antibody at 1:1000 for the heparan sulfate or human Synd1 blots for 1 h at room temperature. The membranes were then washed again three times for 5 min with buffer #3. All membranes were developed using *ECL Plus *Western Blotting Detection kit (Amersham Biosciences, NJ) and Kodak BioMax Light Film (Sigma, MO). The results were quantified by scanning the exposed film, and evaluating the intensity of exposed dots by software AlphaEase FC (Alpha Innotech, San Leandro, CA). Results were expressed as amount of Synd shed in relative absorbance units (AU) using a calibration curve generated by two-fold dilutions of culture supernatants from mouse or human epithelial cells treated with AnlO. The absolute amounts of shed ectodomain varied between experiments, presumably because of the sensitivity of shedding to small variations in treatment conditions noticed previously [5]. Each AU measurement represents the mean and the 95% confidence intervals calculated using the Student t-test.

### Lactate dehydrogenase (LDH) release from treated cells

The LDH activity in the culture medium was measured as index of cytolysis using a spectrophotometric assay with pyruvate and NADH as substrates according to the manufacturer's protocol (Roche Applied Science, Germany) using 10 μl samples of culture medium from treated or untreated cells. To determine total intracellular LDH activity the untreated cells were lysed with 1% Triton X-100, and the assay was performed simultaneously with the test culture medium samples. The fraction of lysed cells in particular treatment conditions was calculated as the LDH release into the incubation medium relative to the total LDH activity present in the epithelial cells before treatment. Each measurement was done in triplicate. The mean and the 95% confidence intervals were calculated using the Student t-test.

### Western blot of Synd1 after heparanase and chondroitinase digestion

Confluent NMuMG cells grown as described above were put in 1% FCS media with either ClnA, Anl B, Anl O or LT for 24 h. After 24 h, the floating cells were removed from supernatant by centrifugation, and proteoglycans in conditioned media were precipitated twice with 4 volumes of 95% ethanol containing 3% potassium acetate. Pellet was resuspended in 100 μM Tris-HCl, pH 8.0 containing 0.1% Triton X-100, 5 μM EDTA, and 1 mM phenylmethylsulfonyl fluoride. Half of each sample was incubated with both 10 mU/ml of heparan sulfate lyase and 25 mU/ml chondroitin sulfate lyase ABC (Seikagaku America Inc., Rockville, MD) for 5 h at 37°C. A fresh portion of enzymes was added after 2.5 h of incubation. Enzyme-treated samples (equivalent to 0.5 ml of conditioned media) were subjected to SDS-PAGE (3.5 to 20% gradient gel) and electro-transferred to Immobilon-N+ membranes (Bio-Rad, CA), which were processed as described above for the dot-blot immunoassay.

### Syndecan-1 and E-cadherin immunostaining

NMuMG cells were grown to confluence on glass slides (BD Falcon and BD BioCoat) for 5 days and then challenged with indicated components. Cells were fixed for 10 min with methanol, washed 3 times with PBS, and then blocked for 20 min with 1% BSA in PBS. After washing with PBS, FITC-labeled murine monoclonal anti-mouse E-cadherin or anti-mouse Synd1 monoclonal antibodies (both from BD Transduction Laboratories) were used for 1 h staining in a dark, after which the slides were washed with PBS, mounted and examined under fluorescence microscope with appropriate filters. Vectashield mounting medium included diamidino phenyl indole (DAPI) for nuclear staining. According to the manufacturer, the anti-mouse E-cadherin antibody cross-reacts with human E-cadherin.

### Analysis of mouse sera after challenge with B. anthracis spores

The 9 week old mice (DBA/2 from Taconic, Germantown, NY) were challenged intraperitoneally with 1 × 10^7 ^spores of *B. anthracis *non-encapsulated Sterne strain 34F2 [pXO1^+^, pXO2^-^] obtained from the Colorado Serum Company (Boulder, CO). The 50% lethaldose of 3 × 10^6 ^spores (LD_50_) by the inraperitoneal (i.p.) route was established earlier [22]. Mice were anesthetized by intraperitoneal injection of Avertin (2,2,2 tribromethanol, Aldrich) at 24 h time points and were bled by cardiac puncture. Serum sample (20 μl) from each mouse was analyzed separately in triplicate with dot blot as described above for cell culture supernatants.

For the ELISA assay of Synd1, serum from each mice (2.5 μl) was diluted in 200 μl of phosphate buffered saline (PBS), 0.5 mM EDTA, 0.1 mM PMSF, 0.1% NP-40 (Pierce) and used to coat wells of the Nunc Maxisorp™ plates (eBiosciences) overnight at 4°C. After incubation, the plates were washed 3 times with 200 μl per well of PBS, 0.1% Tween-20 and blocked for 1 h at 4°C with 200 μl per well of PBS plus 1% BSA (Sigma). Plates were incubated in 100 μl per well of fresh blocking solution plus 1:1000 dilution of rat anti-mouse Synd1 antibody 281-2 (BD Biosciences) for 2 h at 4°C, washed 5 times with 200 μl per well of PBS, 0.1% Tween-20, and finally incubated at room temperature for 1 h with 100 μl per well of goat anti-rat HRP-conjugated secondary antibody (Jackson Immunoresearch) diluted 1:7500 with blocking solution. After incubation, plates were washed 5 times with 300 μl per well of PBS, 0.3% Tween-20 and developed using tetramethyl benzidine reagent (Beckman Coulter) added to all wells and incubated at room temperature for 30 min. Absorbance was detected at 450/570 nm using μQuant plate reader (Bio-Tek Instruments). The average absorbance calculated for each treatment group was corrected for the average absorbance of the control wells without serum, and the results were presented as a fold change relative to the untreated group value. The concentration-response curve was obtained using naïve mouse serum from the unchallenged control group spiked with serial dilutions of recombinant murine Synd1. The curve shows a linear slope (R^2 ^= 0.998) in the tested interval of spiked Synd1 concentrations (0 to 22 μg/ml). It allows estimate the Synd1 release due to infection, however the amount of Synd1 present in normal serum cannot be determined.

### Epithelial barrier permeability studies

HSAECs were grown on commercially available Costar inserts (Corning, NY) consisting of 12 mm permeable PTFE membranes with a pore size of 3.0 μm, and coated by the manufacturer with equal mixtures of Type I and Type III Collagen. Cells were seeded at concentrations of 1 × 10^5 ^cell/ml and allowed to grow till confluence in DMEM/F12 complete medium with 10% FCS. Before challenge the FCS content was reduced to 1%. Epithelial barrier permeability to small molecules was evaluated after the cells were treated with shedding inducers by the addition of Blue Dextran 2000 (Sigma, MO) to the upper chamber of the inserts above the epithelial cells, incubation for 2 h, and then measuring the absorbance of medium in the lower chamber at 600 nm. The absorbance values were normalized relative to the untreated cells control.

### Statistical analyses

Data are presented as means ± confidence intervals for replicate experiments (n ≥ 3) unless indicated otherwise. Unpaired two-tailed Student's t test was used for statistical analyses, and confidence intervals were calculated for P = 0.05.

## Abbreviations

Anthralysin A, AnlA (phospatidyl choline-preferring phospholipase C from *Bacillus. anthracis*); anthralysin B, AnlB (sphingomyelinase from *B. anthracis*); anthralysin O, AnlO (cholesterol-binding pore-forming factor from *B. anthracis*); cereolysin A, ClnA (*B. cereus *homolog of AnlA); heparan sulfate, HS; human small airway epithelial cells, HSAECs; lactate dehydrogenase, LDH; lethal toxin, LT; normal murine mammary gland epithelial cells, NMuMG cells; proteoglycan, PG; syndecan, Synd.

## Authors' contributions

SP conceived the study, developed the research plan, directed experimental work and was principal writer of the manuscript. TP, BM, CBr and SN carried out the experiments and participated in the manuscript writing. CB and VC participated in research coordination and helped to draft the manuscript. All authors read and approved the final manuscript.

## Figures and Tables

**Table 1:** Effect of inhibitors on Synd1 ectodomain shedding from NMuMG cells enhanced by *B. anthracis *proteins

Inhibitors	Shed Synd1 (mean % of no-inhibitor control ± C.I.)				
	
	Spontaneous	ClnA	AnlB	AnlO	LT
**No inhibitor**	100 ± 11	100 ± 9	100 ± 12	100 ± 22	100 ± 1
**Tyrphostin A25**				
5 μM 50 μM	86 ± 6 72 ± 7	100 ± 9	85 ± 17	73 ± 23 67 ± 7	97 ± 8 81 ± 4
**Piceatannol**				
0.5 μM 5 μM 50 μM	191 ± 31 112 ± 24 46 ± 4	17 ± 9	6 ± 2	136 ± 30 88 ± 25 14 ± 9	199 ± 15 79 ± 12 25 ± 2
**Suramin**				
20 μM 200 μM	160 ± 27 36 ± 4	89 ± 17	118 ± 7	155 ± 26 25 ± 4	144 ± 14 34 ± 4
**PD98059**				
5 μM 50 μM	130 ± 10 133 ± 4	103 ± 15	88 ± 3	84 ± 5 56 ± 5	90 ± 4 77 ± 4
**SB202190**				
5 μM 50 μM	97 ± 10 91 ± 10	108 ± 17	101 ± 19	72 ± 1 39 ± 1	69 ± 4 37 ± 5
**JNK inhibitor II**				
1.5 μM 15 μM	125 ± 9 145 ± 9	92 ± 13	129 ± 31	89 ± 5 90 ± 5	109 ± 7 103 ± 3

NMuMG cells in 1% FCS medium were preincubated with the indicated concentrations of inhibitors for 1 h, and then exposed to shedding inducers (1 μg/ml of either ClnA, AnlB or LT; 0.1 μg/ml AnlO) for 24 h. Data are expressed relative to shedding observed without inhibitors in cells either treated or untreated with shedding inducers. Confidence intervals correspond to P = 0.05.
